# Estimating the national burden of hospitalizations for influenza-associated severe acute respiratory infection in the Lao People's Democratic Republic, 2016

**DOI:** 10.5365/wpsar.2020.11.2.001

**Published:** 2021-06-22

**Authors:** Bouaphanh Khamphaphongphane, May Chiew, Joshua A. Mott, Sombandith Khamphanoulath, Viengphone Khanthamaly, Keooudomphone Vilivong, Thongchanh Sisouk, Leila Bell, Erica Dueger, Sheena Sullivan, Angela Daniella Iuliano, Reiko Tsuyuoka, Onechanh Keosavanh

**Affiliations:** a National Center for Laboratory and Epidemiology, Vientiane, Lao People’s Democratic Republic.; b WHO Health Emergencies Programme, World Health Organization, Vientiane, Lao People’s Democratic Republic.; c Influenza Division, Thailand Regional Influenza Program, United States Centers for Disease Control and Prevention, Nonthaburi, Thailand.; d Influenza Division, United States Centers for Disease Control and Prevention, US Embassy, Vientiane, Lao People’s Democratic Republic.; e Health Emergency Information and Risk Assessment, WHO Health Emergencies Programme, World Health Organization, Regional Office for the Western Pacific, Manila, Philippines.; f Infectious Hazards Management, WHO Health Emergencies Programme, World Health Organization, Regional Office for the Western Pacific, Manila, Philippines.; g Influenza Division, United States Centers for Disease Control and Prevention, Atlanta, GA, United States of America.; h Sanofi Pasteur, Lyon, France.; i WHO Collaborating Centre for Reference and Research on Influenza, Royal Melbourne Hospital, Melbourne, and the Peter Doherty Institute for Infection and Immunity, University of Melbourne, Melbourne, Australia.

## Abstract

**Objective:**

Estimates of the burden of influenza are needed to inform prevention and control activities for seasonal influenza, including to support the development of appropriate vaccination policies. We used sentinel surveillance data on severe acute respiratory infection (SARI) to estimate the burden of influenza-associated hospitalizations in the Lao People's Democratic Republic.

**Methods:**

Using methods developed by the World Health Organization, we combined data from hospital logbook reviews with epidemiological and virological data from influenza surveillance from 1 January to 31 December 2016 in defined catchment areas for two sentinel sites (Champasack and Luang Prabang provincial hospitals) to derive population-based estimates of influenza-associated SARI hospitalization rates. Hospitalization rates by age group were then applied to national age-specific population estimates using 2015 census data.

**Results:**

We estimated the overall influenza-associated SARI hospitalization rate to be 48/100 000 population (95% confidence interval [CI]: 44–51) or 3097 admissions (95% CI: 2881–3313). SARI hospitalization rates were estimated to be as low as 40/100 000 population (95% CI: 37–43) and as high as 92/100 000 population (95% CI: 87–98) after accounting for SARI patient underascertainment in hospital logbooks. Influenza-associated SARI hospitalization rates were highest in children aged < 5 years (219; 95% CI: 198–241) and persons aged ^3^ 65 years (106; 95% CI: 91–121).

**Discussion:**

Our findings have identified age groups at higher risk for influenza-associated SARI hospitalization, which will support policy decisions for influenza prevention and control strategies, including for vaccination. Further work is needed to estimate the burdens of outpatient influenza and influenza in specific high-risk subpopulations.

## 

Globally, seasonal influenza is estimated to be associated with severe respiratory illness in 3–5 million people (*1*) and with 290 000–650 000 deaths from respiratory illness each year. (*2*) Although the majority of people infected with seasonal influenza recover, it can cause severe illness or death, particularly in high-risk groups, including pregnant women, children aged < 5 years, older people and individuals with comorbidities. (*1*) In low- and middle-income countries and countries in the tropics, the burden of influenza is poorly understood. (*3*)

In the Lao People's Democratic Republic (Lao People's Democratic Republic PDR), respiratory samples are collected to be tested for influenza at six sentinel sites monitoring severe acute respiratory infection (SARI). Aggregated data at these sentinel sites are also collected by age and sex. At present, SARI sentinel surveillance operates in one central hospital in Vientiane, the capital, and five provincial hospitals that represent the central, northern and southern regions of the country. Influenza viruses have been found to circulate year-round in the country, with typical epidemic peaks from July to December. (*4*) This trend is consistent with trends seen in neighbouring countries with similar environments, such as Cambodia. (*5*)

In 2012, Lao People's Democratic Republic PDR introduced a national seasonal influenza vaccination policy. Since then, the country has implemented this programme through a public–private partnership that offers influenza vaccine to pregnant women, persons aged (*3*)50 years, persons with chronic diseases and health-care workers. Although 90% of health-care workers are currently vaccinated, due to limited availability of the vaccine, coverage is only 35% among pregnant women and 12% among elderly people with chronic conditions. (*6*)

Estimating the burden of people hospitalized with influenza is a key step towards building the evidence base to inform decisions about influenza prevention and control policies. At present, the burden of people hospitalized with influenza is not well understood in the country. Our study aimed to estimate the burden of influenza-associated SARI hospitalizations to inform the evidence base for future decision-making about strategies to prevent and control influenza.

## Methods

We used the World Health Organization (WHO) manual for estimating influenza disease burden (*7*) to identify a method to generate estimates of influenza-associated SARI hospitalizations. Following an assessment of all six SARI sentinel sites, we selected two: Champasack (CPS) Provincial Hospital and Luang Prabang (LPB) Provincial Hospital. We selected these two hospitals because they had catchment areas that were well circumscribed to allow their service populations to be assessed through hospital logbook reviews to obtain denominators for estimating hospitalization rates. These hospitals also represented populations in the northern and southern parts of the country.

### Data sources

#### SARI influenza surveillance system for hospitalized patients

In Lao People's Democratic Republic PDR, patients are identified as having SARI if they have a history of subjective or measured fever of (*3*)38 °C and cough, with onset occurring within the last 7 days, and if they required hospitalization. All patients at the sentinel sites who met the SARI case definition were enrolled in the study, and nasal and throat swabs were collected. The data collected included information on the age, sex and clinical characteristics of the patient. Specimens collected from SARI patients were sent daily to the National Influenza Center at the National Center for Laboratory and Epidemiology in Vientiane where they were tested by real-time reverse transcription–polymerase chain reaction (RT–PCR) for influenza viruses.

#### Health admission data

We reviewed health admission data to estimate the catchment areas of sentinel hospitals and to estimate annual cases of influenza-associated SARI in the country.

#### Estimating the catchment population of sentinel hospitals

At the time of the study, SARI sentinel surveillance in Lao People's Democratic Republic PDR did not capture information about case patients’ district of residence. To determine the catchment areas of the CPS and LPB provincial hospitals, we used data from an unpublished review of all hospital admission records from 2014 (Khampapongpane B, Musto J, Phengxay M, Ketmayoon P, Khamising A, Souphatsone Houatthongkham S, et al., unpublished data, 2017). The catchment area for each hospital was defined as the districts of residence from which (*3*)80% of SARI patients sought care, as guided by the WHO’s manual for estimating the influenza disease burden. (*7*)

**Figure 1:**
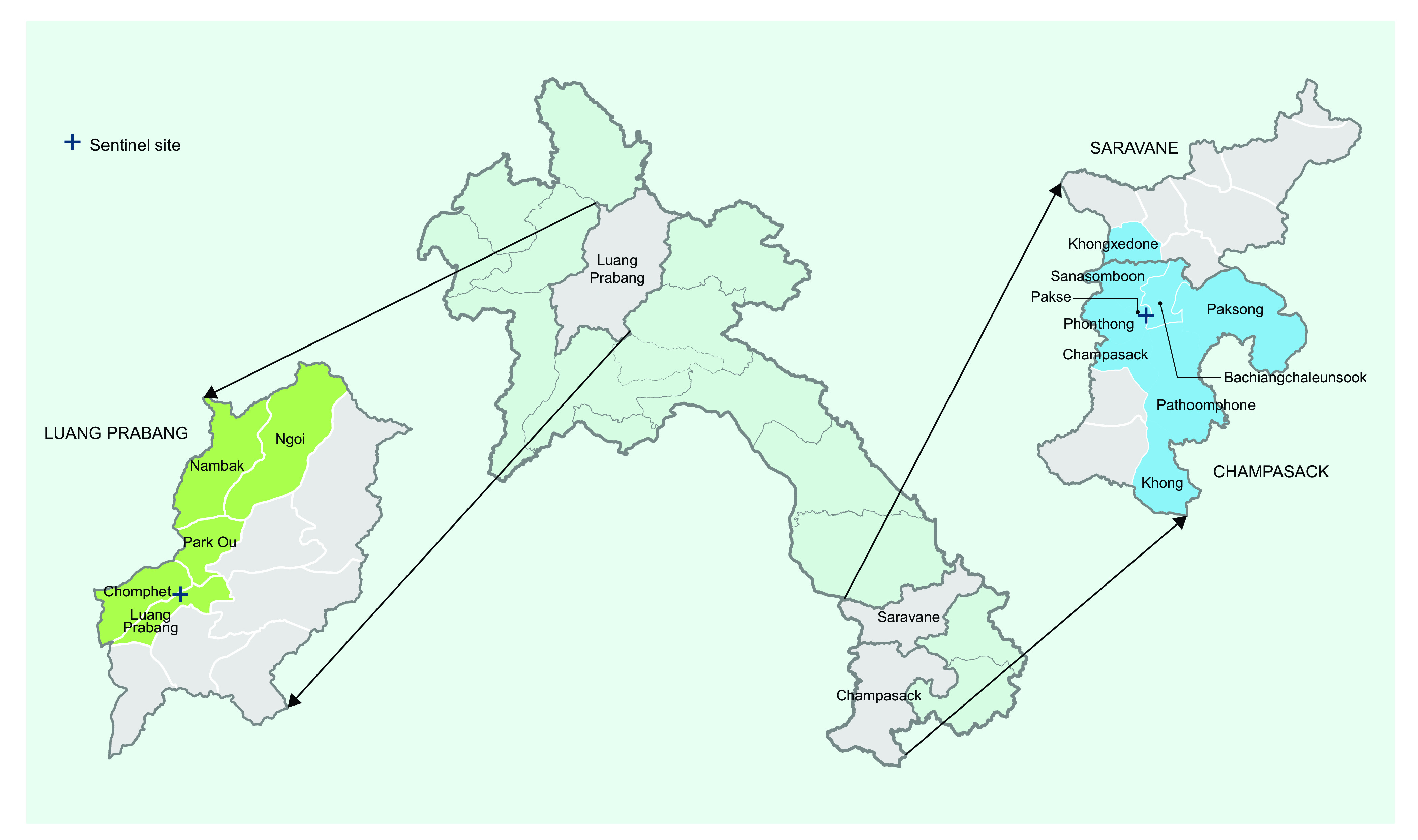
Map of Lao People’s Democratic Republic and the catchment areas of Champasack Provincial Hospital (blue) and Luang Prabang Provincial Hospital (green), by district

The catchment areas for the two sites are shown in **Fig. 1**. The catchment area for CPS Provincial Hospital covered nine districts: eight districts in CPS province (Bachiangchaleunsook, Champasack, Khong, Pakse, Pathoomphone, Paksong, Phonthong and Sanasomboon) and one district in Saravane province (Khongxedone). For LPB Provincial Hospital, the catchment area covered five districts. All of the districts were part of LPB province (Chomphet, Luang Prabang, Nambak, Ngoi and Park Ou). All patients living outside these districts were excluded from the subsequent reviews of hospital admission logbooks.

[**insert** Figure 1]

#### Identifying SARI patients residing within the catchment populations

We visited all health facilities that admit patients with respiratory illness within the identified catchment areas. At those facilities, we obtained and reviewed hospital admission logbooks from wards that admitted patients with respiratory illness (that is, internal medicine, inpatient, intensive care and paediatric units). We included admissions from 1 January to 31 December 2016. In Lao People's Democratic Republic PDR, the International Classification of Diseases, tenth revision (ICD-10), is not used at the subnational level, including at health facilities in the catchment areas. Therefore, we recorded the clinical signs and symptoms from free-text entries in the admission and discharge logbooks to identify SARI patients residing in the catchment areas. Hospitalized patients who met the SARI case definition based on signs and symptoms were considered SARI patients. From the logbooks, we collected demographic data, the dates of admission and discharge, signs and symptoms, onset date, admitting diagnosis, discharge diagnosis and outcomes. To determine the proportion of total inpatient visits that were associated with SARI at LPB and CPS hospitals, we divided the total number of SARI patients identified by the logbook reviews by the total number of inpatient visits at the hospitals during the same period as was recorded in the District Health Information System.

#### Estimating annual cases of influenza-associated SARI, and correcting for missing records

We obtained monthly age-specific numbers of SARI patients from the two sentinel sites and combined them. To account for variation in influenza circulation by month, we then divided the monthly SARI patient counts for each age group by the overall annual percentage of specimens testing positive for influenza at the two sentinel sites because there were too few age-specific data by month. From these calculations, we obtained estimates of the number of cases of influenza-associated SARI by month.

To assess the completeness of the identification of SARI patients in the logbooks, we compared the number of SARI patients detected from prospective sentinel surveillance data in 2016 to the number of SARI patients identified through logbook reviews at both sentinel sites. This was an aggregate-level comparison as it was not possible to link individual patients identified in logbook entries with those identified through SARI sentinel surveillance. Based on pooled results of record reviews from both sites, we calculated a correction factor to account for an underascertainment of SARI patients in the logbook reviews and applied it to the number of patients with influenza-associated SARI by age and month. Due to the absence of links between individual patients in the logbooks and in surveillance data, 95% confidence intervals (95% CIs) could not be estimated for each correction factor. However, as there was variability between the sites in the number of missing logbook records, we also calculated lower- and upper-bound correction factors based on the missing logbook data from each site.

#### Estimating the national burden of influenza-associated SARI

We estimated age-specific populations for each district within the catchment areas using provincial-level age distributions from the 2015 population census (< 5 years: 12.6%; 5 to < 15 years: 23.3%; 15 to < 65 years: 59.8%; and (*3*)65 years: 4.3%). (*8*) We then calculated the adjusted population denominator by multiplying the population of the catchment area of each sentinel site by the proportion of SARI patients that presented to that site compared with other health facilities in the catchment area, by age group. We estimated monthly influenza-associated hospitalizations due to SARI by combining estimated SARI patient counts (the numerator) at the two sites. We divided these combined SARI patient counts by the sum of the adjusted catchment populations for both sentinel sites (the denominator) and multiplied by 100 000. To create annual rates, we aggregated these monthly rates and weighted them by the proportion of SARI patients identified in the logbook review that occurred within a given month of the calendar year.

We adjusted the numbers and rates of influenza-associated SARI hospitalizations by applying the three correction factors for logbook underascertainment. To derive estimates of the national burden, we used the pooled hospitalization rates from the two sentinel site catchment areas and extrapolated these rates to the national 2015 census population. We calculated 95% confidence intervals by applying an error factor, as outlined in the WHO manual, (*7*) to account for variance in the percentage of cases positive for influenza and in the monthly SARI patient counts.

## Results

The hospital logbook review was conducted in 8 of the 12 health facilities in the catchment area of LPB Provincial Hospital (that is, the provincial hospital, one private hospital, one military hospital and five district hospitals) and in 9 of the 10 health facilities in the catchment area of CPS Provincial Hospital (that is, the provincial hospital and eight district hospitals). From January through December 2016, 2060 SARI patients were identified from the review of logbooks, of whom 1513 were from the CPS catchment area and 547 were from the LPB catchment area.

Of the 1513 SARI patients in the CPS catchment area, 823 (54%) were < 5 years; 265 (18%) were 5 to < 15 years; 270 (18%) were 15 to < 65 years; and 155 (10%) were (*3*)65 years. Within this catchment area, 746 (49%) SARI patients were identified from logbooks at CPS Provincial Hospital and 767 (51%) were identified from logbooks at district hospitals. Of the 746 cases identified in the CPS Provincial Hospital logbooks, the median length of stay at the hospital was 2 days.

Of the 547 SARI patients in the LPB catchment area, 313 (57%) were < 5 years; 55 (10%) were 5 to < 15 years; 126 (23%) were 15 to < 65 years; and 53 (10%) were (*3*)65 years. Within this catchment area, 124 (23%) SARI patients were identified from logbooks at LPB Provincial Hospital; 329 (60%) were identified in logbooks at district hospitals; 66 (12%) were identified in logbooks at military hospitals; and 28 (5%) were identified in logbooks at private hospitals. Of the 124 SARI cases identified in the logbooks at LPB Provincial Hospital, the median length of stay at the hospital was also 2 days.

The number of SARI patients identified, the estimated percentage testing positive for influenza by age, the estimated population of the catchment areas and the population denominators are summarized in **Table 1**. Compared with the LPB catchment area, in the CPS catchment area there was a greater number of SARI patients (746 versus 124), a higher percentage testing positive for influenza viruses (18% versus 6%) and a larger adjusted population denominator (325 671 versus 55 850). Based on inpatient data from the District Health Information System, the proportion of inpatient visits due to SARI was approximately 5% (746/15 144) in the CPS Provincial Hospital catchment area and approximately 1% (124/9172) in the LPB Provincial Hospital catchment area.

**Table 1:** Patients with severe acute respiratory illness identified from reviews of hospital logbooks, and catchment area populations, for Champasack Provincial Hospital and Luang Prabang Provincial Hospital, Lao People's Democratic Republic, January to December 2016

Hospital	Age group				Total*
	< 5 years	5 to < 15 years	15 to < 65 years	^3^65 years	
**Champasack Provincial Hospital**					
**Number of SARI patients identified through logbook review**	**344**	**81**	**202**	**119**	**746**
**Percentage of SARI patients positive for influenza at sentinel surveillance site**	**16%**	**34%**	**14%**	**9%**	**18%**
**Population of catchment area**	**78 188**	**140 672**	**411 078**	**30 579**	**660 510**
**Percentage of SARI patients in the catchment area admitted to the sentinel site**	**42%**	**31%**	**75%**	**77%**	**49%**
**Adjusted population denominator**	**32 681**	**42 998**	**307 547**	**23 477**	**325 671**
**Luang Prabang Provincial Hospital**					
**Number of SARI patients identified through logbook review**	**32**	**19**	**43**	**30**	**124**
**Percentage of SARI patients positive for influenza at sentinel surveillance site**	**5%**	**6%**	**9%**	**5%**	**6%**
**Population of catchment area**	**31 067**	**57 404**	**147 329**	**10 569**	**246 370**
**Percentage of SARI patients in the catchment area admitted to the sentinel site**	**10%**	**35%**	**34%**	**57%**	**23%**
**Adjusted population denominator**	**3176**	**19 830**	**50 279**	**5982**	**55 850**

SARI: severe acute respiratory illness.

* Not all row totals equal the sum of columns.

A total of 1253 SARI patients were detected from active sentinel surveillance compared with 870 identified by the logbook reviews, resulting in a correction factor of 1.44 (1253/870). At CPS Provincial Hospital, a total of 908 SARI patients were detected through active sentinel surveillance compared with 746 identified through logbook review. At LPB Provincial Hospital, the numbers of SARI patients identified through prospective surveillance and logbook review were 345 and 124, respectively. Therefore, we also applied the site-specific lower- and upper-bound correction factors for missing logbook data of 1.22 (908/746) and 2.78 (345/124) to the number of SARI patients identified from the logbooks at all hospitals within the catchment areas.

The estimated rate of influenza-associated SARI hospitalization was 48/100 000 population (95% CI: 44–51) (**Table 2**). However, given the variability in SARI patient underascertainment in hospital logbooks, we estimated these overall SARI hospitalization rates to be as low as 40/100 000 population (95% CI: 37–43) and as high as 92/100 000 population (95% CI: 87–98). Our primary pooled incidence rates for the two catchment areas suggested that rates of influenza-associated SARI hospitalization per 100 000 population were highest in children aged < 5 years (219; 95% CI: 198–241). The rates followed a U-shaped curve, declining to 33/100 000 (95% CI: 28–39) for the 5 to < 15 year age group and to 14/100 000 (95% CI: 13–16) for the 15 to < 65 year age group, but increasing to 106/100 000 among persons aged (*3*)65 years (95% CI: 91–121).

**Table 2:** National incidence estimates of rates of hospitalization for severe acute respiratory infection due to influenza in the Lao People's Democratic Republic, by age group, with adjustments to lower and upper bounds for underascertainment of the illness in hospital logbooks, January to December 2016

Age group (years)	Rate of influenza-associated SARI hospitalizations per 100 000 population (95% CI)*		
	Corrected rate	Lower bound of estimate	Upper bound of estimate
** < 5**	**219 (198–241)**	**186 (166–205)**	**423 (390–457)**
**5 to < 15**	**33 (28–39)**	**28 (23–33)**	**64 (56–72)**
**15 to < 65**	**14 (13–16)**	**12 (11–13)**	**28 (25–30)**
**^3^65**	**106 (91–121)**	**90 (76–103)**	**204 (183–226)**
**All ages**	**48 (44–51)**	**40 (37–43)**	**92 (87–98)**

CI: confidence interval; SARI: severe acute respiratory illness.

* The multiplier for case underascertainment was 1.44. The estimate of the lower bounds used a multiplier of 1.22, and the estimate of the upper bounds used a multiplier of 2.78.

Applying these rates to the total population of the country gives the estimated number of influenza-associated SARI hospitalizations in 2016 as 3097 (95% CI: 2880–3313). Accounting for hospital logbook underascertainment, this number was estimated to be as low as 2623 (2431–2816) and as high as 5978 (5625–6331) (**Table 3**). Nearly half of these influenza-associated SARI hospitalizations were estimated to occur in children aged < 5 years.

**Table 3:** National estimated number of hospitalizations for severe acute respiratory infection due to influenza in the Lao People's Democratic Republic, by age group, with adjustments to lower and upper bounds for underascertainment of the illness in hospital logbooks, January to December 2016

Age group (years)	Population of Lao People's Democratic Republic PDR, 2015	Number of influenza-associated SARI hospitalizations (95% CI)		
		Corrected	Lower bound of estimate	Upper bound of estimate
** < 5**	**681 983**	**1496 (1 349–1642)**	**1267 (1 135–1400)**	**2888 (2 661–3115)**
**5 to < 15**	**1 397 815**	**465 (386–545)**	**394 (322–467)**	**898 (784–1013)**
**15 to < 65**	**4 137 333**	**593 (531–654)**	**502 (446–558)**	**1144 (1 052–1236)**
**^3^65**	**275 097**	**291 (250–332)**	**247 (209–284)**	**562 (503–621)**
**All ages**	**6 492 228**	**3097 (2 880–3313)**	**2623 (2 431–2816)**	**5978 (5 625–6331)**

CI: confidence interval; Lao People's Democratic Republic PDR: Lao People's Democratic Republic; SARI: severe acute respiratory illness.

* The multiplier for case underascertainment was 1.44. The estimate of the lower bounds used a multiplier of 1.22, and the estimate of the upper bounds used a multiplier of 2.78.

## Discussion

Our findings are the first to estimate the national burden of influenza-associated SARI hospitalizations in Lao People's Democratic Republic PDR and are important in understanding the health impact of influenza within the country. We found that children aged < 5 years and adults aged (*3*)65 years had the highest rates of hospitalization for influenza-associated SARI.

While every influenza season is different, our results suggest that influenza-associated SARI hospitalization rates for children aged < 5 years in Lao People's Democratic Republic PDR are higher than what has been documented in WHO’s Western Pacific Region. In a recent systematic review and meta-analysis of the global burden of influenza in paediatric respiratory hospitalizations, (*9*) the pooled influenza-associated hospitalization rate among children aged < 5 years was 150/100 000 population (95% CI: 105–216) compared with our estimate of 220/100 000 population. These findings are also similar to the results of a published study in Cambodia that estimated national rates of severe influenza were 323/100 000 population in infants aged < 1 year and 196/100 000 population in children aged 1–4 years. (*10*) In contrast, the incidence of hospitalized patients with acute respiratory infection associated with influenza A in Viet Nam from 2007 through 2008 in children aged < 5 years was much higher, at 870/100 000 population. (*11*) In the Viet Nam study, the case definition included all children presenting with cough or difficulty breathing, or both, with or without fever, (*11*) while our case definition was less sensitive and more specific. However, caution is required in comparing hospitalization rates across countries as case definitions, health-seeking behaviour, admission practices, logbook and medical charting, the methods of calculating population denominators, influenza vaccine policy, the general health of the population and influenza activity vary between countries and over time.

Our estimates suggest that in 2016 influenza represented a significant burden to hospitalizations in Lao People's Democratic Republic PDR. Currently, the government is procuring seasonal influenza vaccine annually, using its own budget, with support from the Partnership for Influenza Vaccine Introduction. (*12*, *13*) These burden estimates will be useful for understanding the impact of influenza by age group. Ongoing work incorporating these estimates is exploring the economic costs of influenza and the cost–effectiveness of influenza vaccines. Understanding the impact of influenza virus infection on the population can support the expansion of influenza vaccine policies in Lao People's Democratic Republic PDR in conjunction with national immunization laws and existing influenza vaccine policies. (*13*) These estimates can also support the government’s decisions to purchase influenza vaccine in the future.

While our estimates will contribute to local and global efforts to estimate the burden of influenza, particularly in Asia, it is also important to acknowledge some limitations. Perhaps most importantly, data from other countries suggest that the SARI case definition used for these analyses may miss a substantial portion of influenza-associated illnesses and may be better suited to virus detection than burden estimation. (*14*) The inclusion of fever in the case definition may be one reason why these estimates are lower than those observed in Viet Nam (*11*) and why only half the burden was seen in children, for whom fever may be a more specific symptom. (*15*) Regardless of the case definition used, prospective sentinel surveillance also will not capture patients in whom an earlier influenza infection may have indirectly caused decompensation of another underlying chronic illness that leads to hospitalization and in whom nucleic acid from influenza viruses can no longer be detected with real-time RT–PCR. This could produce an underestimate of the influenza burden in certain populations, particularly older adults with underlying conditions. (*16*) It was not possible to calculate rates of influenza-associated SARI hospitalization among other recognized high-risk groups, such as pregnant women or patients with underlying conditions, due to the nature of the health systems and because the wards under surveillance in the SARI sentinel system do not necessarily admit those patients.

Because only two sentinel sites served well circumscribed at-risk populations, these data also may not be fully representative of the national population. The variability of missing logbook data, coupled with the absence of ICD-10 coding, also complicated our ability to estimate the national burden of influenza-associated SARI, and these issues created uncertainty about the degree to which missing logbook data impacted these national estimates. We attempted to address this issue using sensitivity analyses.

We are also uncertain of how many people living in the catchment areas travel to other countries, such as Thailand, for medical treatment. Previous studies have demonstrated that people from Lao People's Democratic Republic PDR seek health care in Thailand. (*17*) Furthermore, a study examining the characteristics of Lao People's Democratic Republic nationals seeking health care in Thailand found that from 2009 through 2011, the diagnosis of unspecified pneumonia was one of the top five inpatient conditions for which Lao People's Democratic Republic nationals were treated each year. (*18*) These findings could contribute to our conservative estimates. Given these additional areas of uncertainty, we should note that the 95% confidence intervals presented here (and suggested in the WHO manual) (*7*) account for only random sampling variation and do not account for classification errors and other possible sources of bias.

Indeed, many of the limitations discussed here apply to similar, if not most, national estimates of influenza burden and meta-analyses globally. Notwithstanding, the estimated burden of hospitalizations for influenza-associated SARI in Lao People's Democratic Republic PDR is comparable to those from other countries and highlights the need to maintain and further strengthen influenza surveillance systems. With proper consideration of these data and the case definition used, these findings contribute to understanding the potential impact of influenza in the country. These data can inform prioritization for influenza control and response activities, including vaccination programmes, in Lao People's Democratic Republic PDR when combined with data on the costs of hospitalization, burden, cost of outpatient influenza, and data on vaccine effectiveness and costs.

## References

[1] Troeger CE, Blacker BF, Khalil IA, Zimsen SRM, Albertson SB, Abate D, et al.; GBD 2017 Influenza Collaborators. Mortality, morbidity, and hospitalisations due to influenza lower respiratory tract infections, 2017: an analysis for the Global Burden of Disease Study 2017. Lancet Respir Med. 2019 1;7(1):69–89. 10.1016/S2213-2600(18)30496-X 30553848 PMC6302221

[2] Iuliano AD, Roguski KM, Chang HH, Muscatello DJ, Palekar R, Tempia S, et al.; Global Seasonal Influenza-associated Mortality Collaborator Network. Estimates of global seasonal influenza-associated respiratory mortality: a modelling study. Lancet. 2018 3 31;391(10127):1285–300. 10.1016/S0140-6736(17)33293-2 29248255 PMC5935243

[3] Lee VJ, Ho ZJM, Goh EH, Campbell H, Cohen C, Cozza V, et al.; WHO Working Group on Influenza Burden of Disease. Advances in measuring influenza burden of disease. Influenza Other Respir Viruses. 2018 1;12(1):3–9. 10.1111/irv.12533 29460425 PMC5818353

[4] Khamphaphongphane B, Ketmayoon P, Lewis HC, Phonekeo D, Sisouk T, Xayadeth S, et al. Epidemiological and virological characteristics of seasonal and pandemic influenza in Lao PDR, 2008-2010. Influenza Other Respir Viruses. 2013 5;7(3):304–11. 10.1111/j.1750-2659.2012.00394.x 22716289 PMC5779841

[5] Hirve S, Newman LP, Paget J, Azziz-Baumgartner E, Fitzner J, Bhat N, et al. Influenza seasonality in the tropics and subtropics – when to vaccinate? PLoS One. 2016 4 27;11(4):e0153003. 10.1371/journal.pone.0153003 27119988 PMC4847850

[6] Phengxay M, Mirza SA, Reyburn R, Xeuatvongsa A, Winter C, Lewis H, et al.; Lao PDR Field Epidemiology Training Cohort Team. Introducing seasonal influenza vaccine in low-income countries: an adverse events following immunization survey in the Lao People’s Democratic Republic. Influenza Other Respir Viruses. 2015 3;9(2):94–8. 10.1111/irv.12299 25598475 PMC4353322

[7] A manual for estimating disease burden associated with seasonal influenza. Geneva: World Health Organization; 2015. Available from: https://apps.who.int/iris/handle/10665/178801, accessed 17 February 2021.

[8] Results of Population and Housing Census 2015 [website]. Vientiane: LAOSIS; 2019. Available from: https://laosis.lsb.gov.la/tblInfo/TblInfoList.do, accessed 17 February 2021.

[9] Lafond KE, Nair H, Rasooly MH, Valente F, Booy R, Rahman M, et al.; Global Respiratory Hospitalizations—Influenza Proportion Positive (GRIPP) Working Group. Global role and burden of influenza in pediatric respiratory hospitalizations, 1982–2012: a systematic analysis. PLoS Med. 2016 3 24;13(3):e1001977. 10.1371/journal.pmed.1001977 27011229 PMC4807087

[10] Ieng V, Tolosa MX, Tek B, Sar B, Sim K, Seng H, et al.; Disclaimer; findings and conclusions in this report are those of the authors and do not necessarily represent the official position of the Centers for Disease Control; Prevention. National burden of influenza-associated hospitalizations in Cambodia, 2015 and 2016. Western Pac Surveill Response J. 2018 10 23;9(5) Suppl 1:44–52. 10.5365/wpsar.2018.9.5.011 31832253 PMC6902650

[11] Yoshida LM, Suzuki M, Yamamoto T, Nguyen HA, Nguyen CD, Nguyen AT, et al. Viral pathogens associated with acute respiratory infections in central vietnamese children. Pediatr Infect Dis J. 2010 1;29(1):75–7. 10.1097/INF.0b013e3181af61e9 19907358

[12] Bresee JS, Lafond KE, McCarron M, Azziz-Baumgartner E, Chu SY, Ebama M, et al.; PIVI Partners Group. The partnership for influenza vaccine introduction (PIVI): Supporting influenza vaccine program development in low and middle-income countries through public-private partnerships. Vaccine. 2019 8 14;37(35):5089–95. 10.1016/j.vaccine.2019.06.049 31288998 PMC6685526

[13] Xeuatvongsa A, Mott JA, Khanthamaly V, Patthammavong C, Phounphenghak K, McKinlay M, et al. Progress toward sustainable influenza vaccination in the Lao Peoples’ Democratic Republic, 2012-2018. Vaccine. 2019 5 21;37(23):3002–5. 10.1016/j.vaccine.2019.04.047 31027926

[14] Marcone DN, Durand LO, Azziz-Baumgartner E, Vidaurreta S, Ekstrom J, Carballal G, et al. Incidence of viral respiratory infections in a prospective cohort of outpatient and hospitalized children aged £5 years and its associated cost in Buenos Aires, Argentina. BMC Infect Dis. 2015 10 24;15(1):447. 10.1186/s12879-015-1213-4 26497393 PMC4619328

[15] Hirve S, Chadha M, Lele P, Lafond KE, Deoshatwar A, Sambhudas S, et al. Performance of case definitions used for influenza surveillance among hospitalized patients in a rural area of India. Bull World Health Organ. 2012 11 1;90(11):804–12. 10.2471/BLT.12.108837 23226892 PMC3506409

[16] Gordon A, Reingold A. The burden of influenza: a complex problem. Curr Epidemiol Rep. 2018;5(1):1–9. 10.1007/s40471-018-0136-1 29503792 PMC5829127

[17] Bochaton A. Cross-border mobility and social networks: Laotians seeking medical treatment along the Thai border. Soc Sci Med. 2015 1;124:364–73. 10.1016/j.socscimed.2014.10.022 25454637

[18] Charoenmukayananya S, Sriratanaban J, Hengpraprom S, Trarathep C. Factors influencing decisions of Laotian patients to use health care services in Thailand. Asian Biomed. 2014;8(5):665–71. 10.5372/1905-7415.0805.342

